# Integrated Study of Polyethylene Microplastic Degradation and Its Interaction with the Insecticide Imidacloprid: Chronic Toxicity to *Daphnia similis*

**DOI:** 10.1007/s00244-026-01213-5

**Published:** 2026-09-02

**Authors:** Maria Eduarda Kiihl, Thandy Junio da Silva Pinto, Mariana Amaral Dias, Cassiana Carolina Montagner, Evaldo Luiz Gaeta Espíndola, David Silva Alexandre

**Affiliations:** 1 Laboratory of Ecotoxicology and Applied Ecology, Department of Hydraulic and Sanitation, São Carlos School of Engineering, University of São Paulo, Av. Trabalhador São Carlense, 400, São Carlos, SP 13560-970 Brazil; 2 Institute of Chemistry, University of Campinas (UNICAMP), Campinas, São Paulo, Brazil

## Abstract

The presence of microplastics (MPs) in freshwater environments represents a growing environmental concern due to their persistence and ability to interact with contaminants such as pesticides. This study evaluated the influence of photodegradation on the toxicity of polyethylene microplastics (PE-MPs), alone and combined with the pesticide Imidacloprid (IMI), on the cladoceran *Daphnia similis*. Microparticles of polyethylene were aged under UV-C radiation for 94 days and subsequently characterized by Scanning Electron Microscopy (SEM) and Fourier-Transform Infrared Spectroscopy (FTIR). Surface analysis indicated polymer degradation through cracks and cavities, and increased surface roughness, while FTIR analyses showed polymer oxidation. Ecotoxicological results revealed that pristine MPs significantly reduced survival by 90% at 80 mg L^−1^, while photodegraded MPs caused more pronounced sublethal effects, particularly on reproduction, with a 69.8% reduction in neonates at 140 mg L^−1^. When associated with IMI, significant reductions in reproduction were observed, with a decrease of up to 75.7% in the number of neonates. However, the presence of IMI attenuated the toxicity of PE-MPs, evidencing an antagonistic interaction between the contaminants, particularly at concentrations of 80 and 140 mg L^−1^ of pristine PE-MPs. Taken together, these results demonstrate that the interactions between MPs and pesticides, as well as the state of particle degradation, significantly modulate ecotoxicological effects, posing a potential risk to the ecological integrity of aquatic ecosystems.

**Supplementary Information:**

The online version contains supplementary material available at https://doi.org/10.1007/s00244-026-01213-5.

## Introduction

The widespread use of plastics in contemporary society, coupled with the exponential increase in their production and consumption over recent decades, has generated growing concern in the scientific community, especially due to the potential impacts to ecosystems and human health (Zhang et al. 2024a). Global plastic production reached 414 million tons in 2023, with an annual growth rate of 5.8% since 2009 (Plastic Europe 2025). Studies have reported that the increase in plastic production has been reflected in the growing occurrence of plastic in ecosystems over the years (Goldstein et al. 2012) As a result, the presence of plastic waste has intensified across environmental compartments, including freshwater environments, among others, in the form of microplastics (MPs), which are classified as particles ranging from 1 μm to 5 mm (Duis and Coors 2016; Olivatto et al. 2018; Eerkes-Medrano et al. 2015; Montagner et al. 2021). Such particles enter water bodies through multiple pathways, such as the discharge of effluents from treatment plants, urban and agricultural runoff, in addition to the progressive fragmentation of plastics (Browne et al. 2011; Horton et al. 2017). Monitoring studies have documented the widespread occurrence of MPs in river catchments, with reported concentrations ranging from 1.9 to 330 items m^−3^ (de Faria et al. 2021; Dikareva and Simon 2019; Ferraz et al. 2020; Rani-Borges et al. 2022). Similarly, in the Elbe River in Germany, considered one of the rivers with the lowest level of contamination, the average observed was 5700 particles m^−3^ (Scherer et al. 2020). In North America, McCormick et al. (2014) found concentrations between 1900 and 17,900 particles m^−3^ in the Chicago River.

A particular concern regarding the widespread presence of MPs in freshwater environments, beyond their direct adverse effects on biota, is their capacity to adsorb chemical contaminants, which may subsequently be transferred to organisms and propagated through the food chain (Rosenkranz et al. 2009; da Silva et al. 2022). The nature of these interactions is intensified by the photodegradation of MPs, a process mediated by exposure to light, which causes the breakdown of polymeric chains and their oxidation, resulting the formation of polar functional groups, such as carbonyls and hydroxyls, which increase their active surface area and favor the adsorption of organic pollutants, including pesticides (Fotopoulou and Karapanagioti 2012; Gewert et al. 2015; Kumanayaka et al. 2010).

Pesticides, widely used in agriculture to control pests and diseases, are a group of contaminants of growing concern in aquatic environments (Kalyabina et al. 2021). The intensive and often inappropriate use of these substances, combined with poor agricultural management practices, favors the entry of compounds such as the insecticide imidacloprid (IMI) into water bodies through spray drift, surface runoff, and leaching (Soares and de Souza Porto 2012; Van Dijk et al. 2013).

Imidacloprid (IMI) is a systemic insecticide belonging to the class of neonicotinoids, widely used in the control of insects, whose mechanism of action is based on interference in their central nervous system, acting as an agonist of nicotinic acetylcholine receptors (nAChR) (Morrissey et al. 2015). It is a highly water-soluble compound (613 mg L^−1^ at 20 °C), with low volatility (vapor pressure of 4 × 10^−10^ Pa at 20 °C), low to moderate affinity for soil and sediment organic matter (Koc ranging from 109 to 411) and moderate lipophilicity (log Kow of 0.57 at 21 °C) (EFSA 2008). In the aquatic environment, IMI presents high stability to hydrolysis at pH conditions between 5 and 9 and temperature of 25 °C, but is susceptible to degradation by photolysis, with half-life values (T50) ranging from 0.2 to 10 days (EFSA 2008).

IMI has been detected in aquatic environments at concentrations ranging from 0.001 to 320 µg L^−1^ (Morrissey et al. 2015), with high toxic potential and the ability to cause adverse effects on the survival, reproduction and behavior of aquatic organisms, such as *D. magna* (Van Den Brink et al. 1997; Rico et al. 2018). Tessaro (2023) recorded high mortality rates in *D. similis* (58–67%) after exposure to IMI concentrations ranging between 1.25 and 5.02 mg L^−1^. Corroborating these findings Alexandre et al. (2025a), found that a concentration of 3.01 mg L^−1^ was sufficient to cause 50% mortality in *Ceriodaphnia silvestrii*, demonstrating the high sensitivity of these microcrustaceans to the contaminant.

This scenario becomes even more worrying when considering the interaction between pesticides and MPs, given the potential of IMI for bioaccumulation and trophic transfer within aquatic food webs (Crayton et al. 2020). Li et al. (2021) demonstrated that polyethylene microplastics (PE-MPs) have a high affinity for IMI molecules, being a potential vector of this insecticide in the environment. Similarly, Mathi (2018) found that the combination of polypropylene MPs with the pesticides 2,4-D and fipronil increased toxicity to *Daphnia similis*, evidencing synergistic effects. This association, according to Rochman et al. (2013), intensifies ecotoxicological risks, especially for invertebrates, since MPs can carry compounds such as insecticides, increasing their bioavailability and promoting greater accumulation in the tissues of aquatic organisms, which can compromise essential ecosystem functions and reduce local biodiversity (Rummel et al. 2017).

In this context, the present study investigated the photodegradation of PE-MPs and characterized the aged particles. It further assessed the ecotoxicological effects of both pristine and photodegraded forms on the cladoceran *D. similis*, tested individually and in combination with insecticide IMI. It was hypothesized that isolated PE-MPs may trigger direct physical effects, such as intestinal tract obstruction, resulting in more pronounced lethal effects, while the presence of IMI in the mixture with PE-MPs may reduce feeding capacity and induce physiological stress, with potential ecological implications in prolonged exposures.

## Materials and Methods

### Polyethylene Microplastics 

White polyethylene microplastics (PE-MPs), with a commercial diameter ranging from 35 to 48 μm and a density of 0.94 g/mL at 25 °C, were purchased from Sigma-Aldrich^®^ Brazil (product no. 434272). The concentrations selected for the experiments were 20, 80, and 140 mg L^−1^ based onCastro et al. (2022), corresponding to 1, 4, and 7.5 times the maximum concentration of MPs reported in freshwater ecosystems (Jambeck et al. 2015; Koutnik et al. 2021). Units were converted from mg L^−1^ to particles^−3^ as described by Leusch and Ziajahromi (2021), resulting in the specifications presented in Table S1. For toxicity tests, MPs were weighed using a precision analytical balance (Bel Engineering model, error 0.0001 g).

The morphological characterization of particles (pristine and aged) was performed by scanning field emission electron microscopy (SEM-FEG), using the Quanta FEG 250 equiMPent (FEI Co. USA). The samples were fixed on conductive carbon tape and coated with a thin layer of evaporative carbon using the Bal-Tec MD020 equipment (Balzers). Particle measurements were performed using Fiji software (Schindelin et al. 2012). The samples were mounted on a conductive carbon ribbon, followed by iridium sputtering coating on a Bal-Tec MD020 instrument (Balzers). Chemical characterization was performed by Fourier transform infrared spectroscopy (FTIR, Agilent Cary 630, Santa Clara, USA), with the attenuated total reflection (ATR) technique, using a diamond crystal in the spectral range of 4000–400 cm^−1^, 64 scans, and resolution of 4 cm^−1^. Analyses were carried out at the Institutional Microscopy Laboratory of the State University of Campinas (UNICAMP).

## PE-MPs Photodegradation

The pristine PE-MPs were photodegraded in an accelerated aging chamber by exposure to UV-C radiation at a wavelength of 240–270 nm (Philips TUV 15 W/G15T8 lamp, Brazil) for 94 days. Samples were positioned 10 cm above the light source at room temperature. To better understand the process in a natural environment, a correlation was established between the time of photodegradation in the laboratory and the degradation under natural conditions, following Eq. (1) according to the method described by Doğan (2021) The UV incidence from solar radiation (400 lx) was estimated based on Liu et al. (2009) and Tyblewski et al. (1991). Additionally, the Philips TUV 15 W/G15T8 lamp used in the experiment presented a luminous intensity of 1140 lx.

1 $$ t_{{real}} = \frac{{I_{l} ~ \times ~t_{{l,~day}} ~}}{{I_{s} ~ \times ~t_{{s,~day}} }}~ \times ~t_{{l,~total}} $$

where:

- t_real_: estimation of degradation time in real environment (days)
- I_l_: lamp illumination dose (lux)
- t_l, day_: exposure time in one day (hours)
- I_s_: incident solar illumination Dose (lux)
- t_s, day_: solar incidence time in a day (hours)
- t_l, total_: time of total light exposure to UV radiation by photobleaching (days)

## Tests Organisms

*Daphnia similis* was obtained from cultures maintained at the Laboratory of Ecotoxicology and Applied Ecology (LEEA) of the University of São Paulo. The cultivation was conducted according to the ABNT NBR 12,373 (2016), under controlled temperature of 20 ± 2 °C and photoperiod of 16:8 h (light: dark). The organisms were kept in reconstituted water prepared from deionized water, supplemented with potassium chloride, calcium sulfate, and magnesium sulfate, adjusted for a total hardness between 40 and 48 mg CaCO_3_ L^−1^, pH 7.0–7.6, and electrical conductivity of 150–160 µS cm^−1^. Before using, the water was aerated for 24 h to stabilize oxygen levels. Feeding was carried out three times a week, using a suspension of the microalgae *Raphidocelis subcapitata*, adjusted according to cell density (2,0 × 10^5^ cells/mL), complemented with 1 mL of fermented fish food (TetraMin^®^). The sensitivity of the cultures was evaluated monthly with potassium chloride.

## Chronic Exposure

The toxicity tests with *D. similis* were conducted using pristine and photodegraded MPs, alone and in mixture with the insecticide IMI. The exposure scenarios comprised seven treatments: ^(1)^ control; ^(2)^ control + acetone (0.01% acetone); ^(3)^ pristine PE-MPs at 20, 80, and 140 mg L^−1^; ^(4)^ photodegraded PE-MPs at 20, 80, and 140 mg L^−1^; ^(5)^ IMI only (1.5 mg L^−1^) ^(6)^ pristine PE-MPs at 20, 80, and 140 mg L^−1^ combined with 1.5 mg L^−1^ IMI; ^(7)^ photodegraded PE-MPs at 20, 80, and 140 mg L^−1^ combined with 1.5 mg L^−1^ IMI. The assays lasted 21 days and were performed on glass breakers, containing 100 mL of the test solution, with one neonate (< 24 h) per container and ten replicates per treatment, according to standard 211 of OECD (2012).

The test solutions for pristine and photodegraded PE-MPs, in concentrations previously described, were initially obtained by dispersing the MPs in a solution containing acetone to reduce the surface tension between the particles and water. Thus, MPs were dispersed in reconstituted water until desired concentrations with a maximum acetone proportion of 0.01%, as recommended by OECD 202 (OECD, 2004). To IMI-only treatment ^(5)^, the test concentration of 1.5 mg L^−1^ was prepared by diluting a stock solution (100 mg L^− 1^) with culturing water. In mixture treatments ^(6,7)^, IMI was first diluted in the test medium to the desired concentration, and the MPs were subsequently dispersed, as previously described.

The test solution was renewed three times a week, at which time the organisms were fed as described to cultures. During each renewal, the survival of females was recorded, and the produced neonates were counted and discarded, allowing the calculation of the reproductive rate. The temperature was kept at 20 ± 2 °C, under a photoperiod of 16:8 h (light: dark). The physicochemical parameters of the water, namely, pH (Micronal B374 pH meter), dissolved oxygen (YSI 55 − 25 oximeter), and electrical conductivity (Orion 145 A conductivity meter) were monitored at the beginning and end of the experiment.

## Chemical Analysis of Imidacloprid

A commercial formulation of imidacloprid, Imidagold 700WG^®^, containing 700 g kg^−1^ of the active ingredient, IMI, was used to prepare the stock solution in reconstituted water. Prior to analysis, all pesticide solutions were filtered through a hydrophobic polytetrafluoroethylene (PTFE) syringe filter (13 mm × 0.22 μm; Analítica, Brazil).

Analytical measurements were performed using a Thermo Vanquish Flex liquid chromatograph coupled to a Thermo TSQ Fortis mass spectrometer (LC-MS/MS). For each sample, 10 µL was injected and eluted using a mobile phase consisting of 95% ultrapure water with 0.1% formic acid (A) and 5% methanol (B). The proportion of solvent B increased to 20% over 3 min, then to 60% in the following 3 min, reaching 80% in 2 min, before returning to 5% in the final 8 min, yielding a total run time of 16 min. The flow rate was set at 0.2 mL min^−1^, and the column temperature was maintained at 30 °C. Chromatographic separation was achieved using a Zorbax SB-C18 column (2.1 × 30 mm, 3.5 μm particle size).

The mass spectrometer operated in positive ionization mode, with a capillary voltage of 3500 V, source fragmentation of 15 V, sheath gas at 35 a.u., auxiliary gas at 10 a.u., and both the ion transfer tube and vaporizer maintained at 350 °C. Ultra-high-purity argon (99.999%) was used as the collision gas at a pressure of 1.5 mTorr. Quantification of analytes was performed in selected reaction monitoring (SRM) mode using the following precursor → product ion transitions and collision energies (eV): IMI 256.0 → 175.0 (18 eV), 256.0 → 209.0 (15 eV), 256.0 → 210.0 (9 eV). Analyte concentrations were determined using external calibration curves ranging from 1.0 to 500.0 µg L^−1^, and data processing was conducted with Thermo Xcalibur software (version 4.7.69.37). The limit of quantification (LOQ) was defined as a signal-to-noise ratio greater than 10, corresponding to 1.0 µg L^−1^.

### Data Analysis

The size distribution of the PE-MPS was represented in histograms (Fig. S2), and statistical analyses were performed using Origin software (version 9.95). The normality and homoscedasticity of the data were evaluated using the Shapiro–Wilk and Levene tests, respectively. Differences in the mean particle size between pristine and photodegraded PE-MPs were assessed using an independent Student’s t-test, based on the mean particle size, standard deviation, and the number of particles measured for each group. The differences between the treatments, considering the survival and reproduction endpoints, were examined using Generalized Linear Models (GLM), allowing statistical comparison between the experimental groups. Additionally, to evaluate the influence of photodegradation on treatments with PE-MPs alone and on exposure combined with IMI a two-way analysis of variance (ANOVA) was employed, considering the concentration of PE-MPs and the degradation status as factors.

Mixture interactions were evaluated by comparing predicted and observed effects, estimated from the responses obtained in single and mixture treatments under each PE-MPs degradation condition, following the approach described by Gottardi et al. (2017). These values were applied to the Model Deviation Ratio (MDR) method, originally proposed by Belden et al. (2007). This method allows for the evaluation of the magnitude and significance of the deviation between the observed effects for the mixture and those predicted from the data obtained for the compounds evaluated individually, based on statistical permutation procedures. In this analysis, the interpretation thresholds established by Cedergreen (2014) were adopted, according to which MDR values < 0.5 indicate antagonistic interactions, 0.5 < MDR < 2 characterize additive responses, and MDR > 2 indicate synergistic interactions. Additionally, the statistical significance of the deviations was evaluated by comparing the observed and predicted effects, allowing verification of whether the detected interactions differed significantly from the reference additive model. All analyses were performed considering a 95% confidence interval (*p* < 0.05), using the Jamovi software (version 2.6.26).

## Results and Discussion

### PE-MPs Characterization and Degradation

The Shapiro-Wilk normality test proved that the particle size data was not normal (*p* < 0.05). The Gaussian model described the histogram of particle size distribution (R^2^ = 0.7749). The morphological characterization revealed circular-shaped particles. To pristine MPs, size characterization showed diameters ranging from 7.2 to 64.8 μm (*n* = 582 particles), with an average of 27 ± 10 μm and a median of 25.2 μm, most frequent measuring 20–25 μm (23.7%), 25–30 μm (22.3%), and 15–20 μm (18.9%). Photodegraded PE-MPs particles presented a particle size range from 3.6 μm to 67.3 μm (*n* = 630), with an average of 30 ± 13 μm and a median of 26.6 μm (Fig. S1 and S2). A statistically significant difference in particle size was observed between pristine and photodegraded PE-MPs (Student’s t-test, *p* = 6.7 × 10^−6^), with photodegraded particles exhibiting a slightly higher mean diameter. This result indicates that the photodegradation process modified the particle size distribution under the experimental conditions tested.

**Fig. 1:**
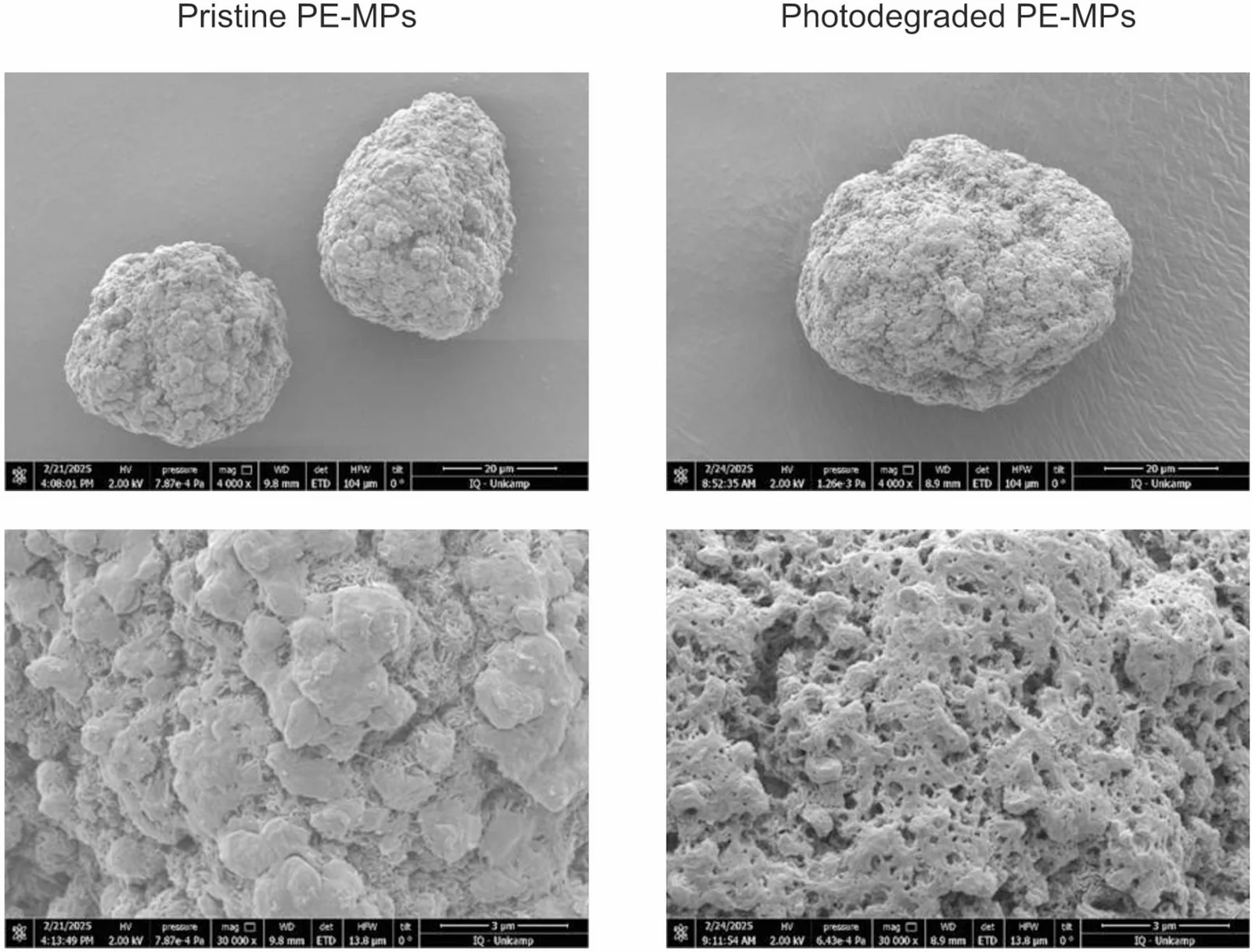
SEM images for pristine and photodegraded PE-MP at scales of 20 μm and 3 μm

SEM images (Fig. 1) showed homogeneous polyethylene particles for pristine and photodegraded MPs at a scale of 20 μm. However, in photodegraded MPs, the surface of the particles is coated with cavities and cracks, indicating a more porous structure that increases roughness, which is most evident at 3 μm and 1 μm scales. These morphological changes reflect the progressive structural weakening of the particles induced by photodegradation, a process that also drives chemical and mechanical alterations in the polymer matrix under UV radiation (Luo et al. 2021; Meides et al. 2021).

**Fig. 2:**
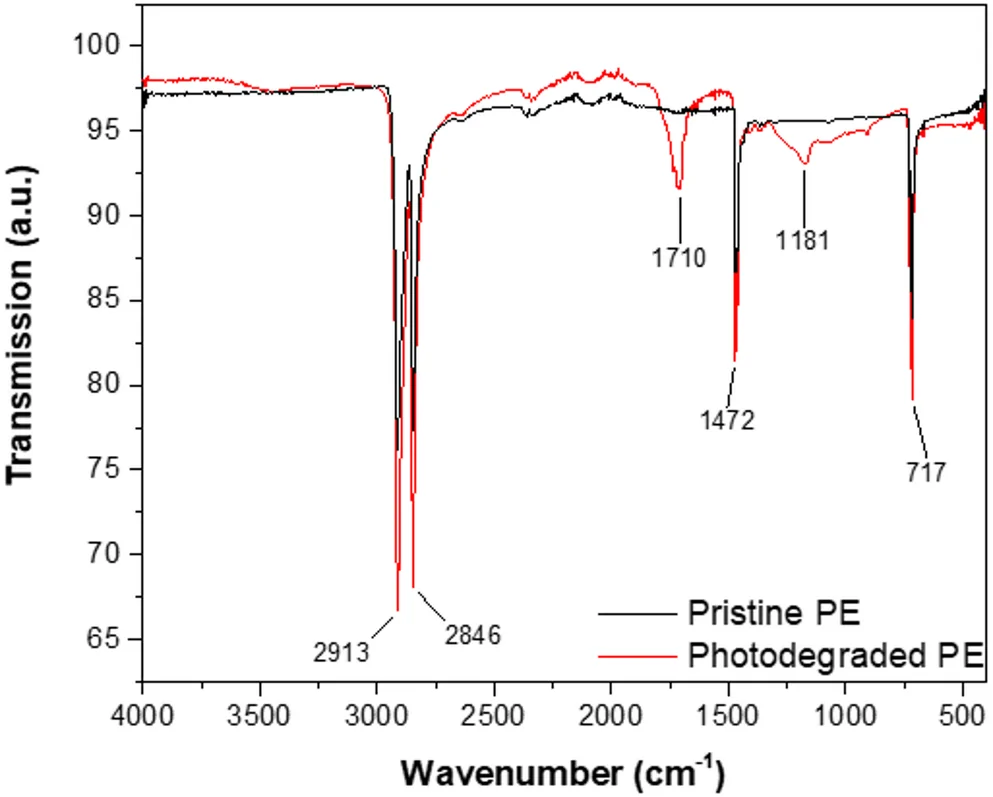
FTIR spectra of pristine and photodegraded PE-MP

The FTIR spectra (Fig. 2) demonstrated the characteristic bands for PE-MPs, where 2846 cm^−1^ and 2913 cm^−1^ correspond to the CH section, 1472 cm^−1^ corresponds to the CH_2_ curve, and 717 cm^−1^ corresponds to the CH_2_ vibration. The bands formed only in aged PE-MPs at 1710 cm^−1^ and 1181 cm^−1^, corresponding to stretches C=O and C–O, respectively. This indicates the oxidation of the polymer surface by the formation of carbonyl groups and secondary alcohol, thus demonstrating its degradation (Jung et al. 2018; Tiago et al. 2025). These oxygenated groups are typical products of polyethylene photo-oxidation, resulting from the abstraction of hydrogens from the methylene₂ group, formation of peroxide radicals, and subsequent breakdown of polymeric chains(Andrady 2011; Li et al. 2022). These findings are consistent with the results of Samir et al. (2026), who observed in low-density polyethylene exposed to UV-C radiation, the appearance of a peak at 1713.9 cm^−1^, attributed to the formation of carbonyl-containing groups, such as ketones, aldehydes, and carboxylic acids.

In the present study, the exposure of PE-MPs to UV radiation in an accelerated weathering chamber, with a light dose equivalent to approximately 643 days of exposure to solar radiation under ambient conditions, evidenced the progression of photodegradation in multiple dimensions. The degradation mechanism identified is consistent with the classical model of polyolefin photodegradation. UV-C radiation provides sufficient energy to induce the formation of free radicals, while the controlled temperature of the chamber, 35–55 °C (Fig. S3), increases the mobility of the chains, accelerating the oxidation process. The accumulation of carbonyls observed in our FTIR spectra corroborates recent studies that reported a similar phenomenon in PE subjected to accelerated aging (Zhang et al. 2024b). These functional groups act as chromophores, increasing the polymer’s susceptibility to new photo-oxidation cycles and intensifying the degradation process over time (Liu et al. 2019).

The chemical modifications observed showed by structural transformations evident in the SEM analyses. While the pristine MPs had a homogeneous and smooth surface, the photodegraded particles exhibited cracks, cavities, and increased roughness, indicating embrittlement and greater porosity of the polymeric matrix. These changes are in line with the two-stage degradation model proposed by Meides et al. (2021) for polystyrene, in which the initial phase of surface photo-oxidation evolves into microcrack formation and fragmentation, accelerating breakdown into smaller particles. In our case, the results suggest that the PE follows a similar dynamic, with a strong link between the initial chemical modifications and the subsequent morphological transformations.

In addition to the direct influence of UV radiation, other factors also modulate the speed and extent of photodegradation. The intensity of radiation, the presence of oxygen, and thermal variation are crucial determinants, as they control the rate of free radical formation and the propagation of oxidation reactions (Gewert et al. 2015; Luo et al. 2021). In this study, even under controlled laboratory conditions, degradation occurred consistently, indicating that, in natural environments, prolonged exposure and greater variability of environmental factors can further intensify PE-MPs fragmentation.

The consequences of these transformations are significant for the properties of the polymer. The insertion of oxygenated groups increases surface polarity, potentiating interactions with organic and inorganic contaminants (Liu et al. 2019). At the same time, the loss of structural integrity, evidenced by cracks and cavities, reduces mechanical strength, favoring fragmentation into smaller particles and the formation of nanoplastics (Amin et al. 1995).

### Effects of Single Exposure to Microplastics

The physicochemical parameters of the water measured at the beginning and end of the experiment remained stable, with electrical conductivity between 151 and 160 µS cm^−1^, pH ranging from 7.0 to 7.3, and dissolved oxygen between 7.1 and 7.4 mg L^−1^. In the controls, the survival rate of *D. similis* was 90% after 21 days, while the average number of neonates produced by surviving females reached 65.9 ± 2.6 individuals. These values fully meet the validity criteria established by the OCDE (2012), which stipulate a maximum mortality of 20% and a minimum production of 60 neonates per surviving female, ensuring the reliability of the tests performed.

**Fig. 3:**
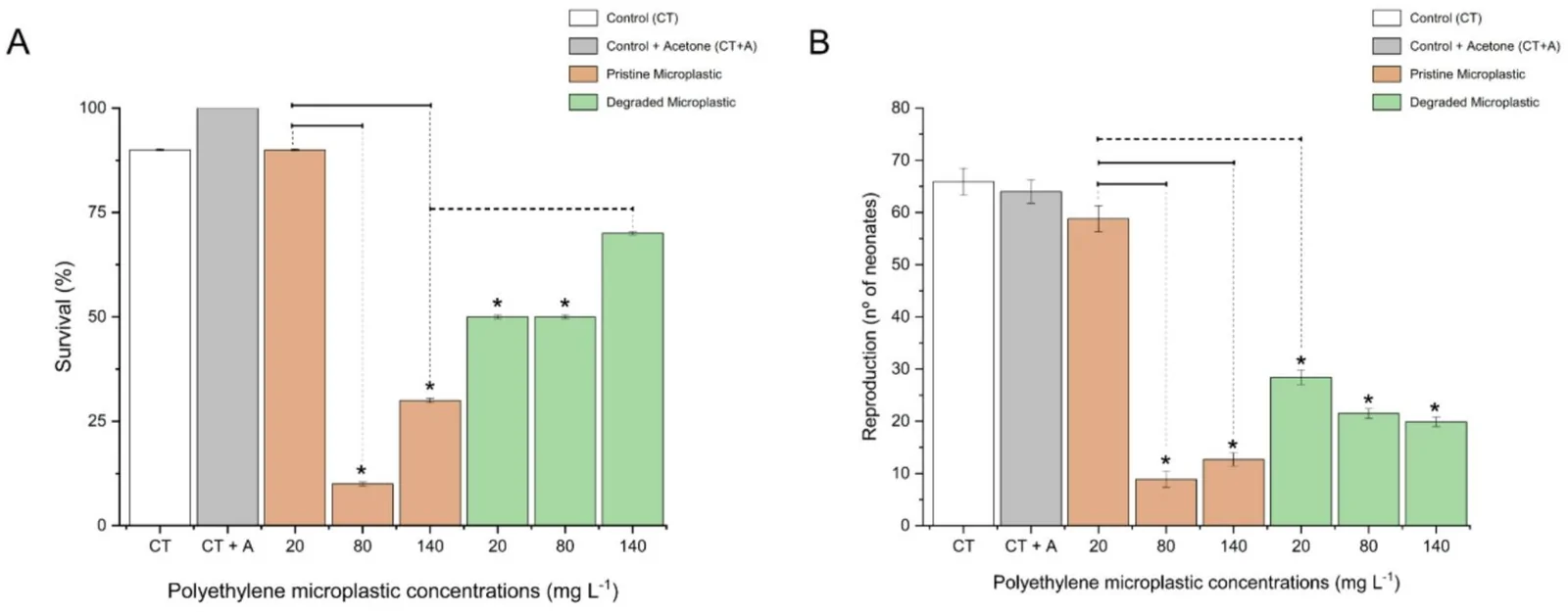
Survival and reproduction with isolated polyethylene microplastic (PE-MPs). Being, CT, control; CT + A, control with 0.01% acetone. (*) Indicates a significant difference compared to the control treatment (*p* < 0.05). Continuous lines indicate significant differences between the concentrations evaluated within the same treatment (*p* < 0.05), whereas dashed lines indicate significant differences between treatments at the same concentration (*p* < 0.05)

Figure 3 shows the survival and reproduction of *D. similis* at each concentration of isolated PE-MPs, pristine and degraded, respectively. Exposure to intermediate (80 mg L^−1^) and high (140 mg L^−1^) concentrations of pristine PE-MPs exerted severe effects on survival, resulting in 90% and 70% mortality, respectively, after 21 days of exposure. These results indicate that the toxicity of pristine PE-MPs does not necessarily follow a linear pattern in which higher concentrations necessarily lead to proportionally greater effects. Rather, the results suggest that concentration influences toxicity, but the observed responses are not monotonic or progressively related to increasing MP concentration. Instead, the impacts appear to manifest at critical concentration levels, at which the physiological tolerance of the species and the bioavailability of the particles determine the magnitude of the effects (Agathokleous et al. 2021). This analysis is supported by Rist and Hartmann (2018), who evaluated the effects of different concentrations of MPs on *D. magna* and observed that the impacts are not distributed proportionally to the dose, but tend to arise when the ingested particle load exceeds the organism’s processing and excretion capacity, intensifying the adverse effects at higher concentrations.

A distinct pattern was observed for the photodegraded MPs evaluated separately, since the most significant reductions in survival occurred in the 20 and 80 mg L^−1^ treatments, both with decreases of 44.4% in relation to the control. This behavior is in line with the results of Serra et al. (2025), who reported significant reductions in the survival of *D. magna* chronically exposed to polyethylene terephthalate (PET) particles and fibers, showing that prolonged exposure promotes cumulative effects capable of compromising population viability. Similarly, de Castro et al. (2023) observed mortality rates between 40% and 80% in *D. similis* exposed for 21 days to PET concentrations (< 53 μm) between 10^5^ and 10^6^ L^−1^ particles, reinforcing the role of exposure conditions as key modulators of toxicity.

The observed reductions in survival are predominantly associated with possible physical effects of MPs. Evidence of ingestion supports this interpretation.(Castro et al. 2020) demonstrated ingestion of PE particles after 24 h across all tested concentrations (20, 40, 80, 160, and 320 mg mL^−1^), with sizes ranging from 40 to 48 μm. Similarly, (Canniff and Hoang 2018) confirmed ingestion of MPs by *Daphnia magna* in the 63–75 μm range. These observations align with the known feeding capacity of cladocerans, which naturally ingest particles ranging from 0.7 to 70 μm algal cells (Burns 1968; Goulden and Hornig 1980). In the present study, indications of the possible presence of MPs in the digestive tract were also observed (Fig. S4), further supporting direct organism–particle interactions.

The presence of these particles in the intestinal tract can induce a false sense of satiety, reducing food intake, compromising the assimilation of microalgae and causing mechanical injuries to the intestinal walls, which directly affects the energy balance and physiological performance of organisms (Lei et al. 2018). Additionally, the retention of MPs in the filtering device can hinder the diffusion of oxygen, favoring the occurrence of hypoxia (Martins and Guilhermino 2018), a condition that tends to intensify the adverse effects on the growth and survival of *D. similis.*

In addition, the higher mortality observed in this study compared to that found in literature can be attributed to the size of the MPs evaluated. When characterizing the PE-MPs, it was found that the mean diameter was smaller than that indicated by the manufacturer, which can be reflected in the results reported here. Previous evidence confirms this trend, indicating that small MPs significantly compromise the survival of *D. magna* when compared to larger particles (Kokalj et al. 2018; Chen et al. 2022). ConsistentlyAlexandre et al. (2025b) exposed the aquatic amphipod *H. meinerti* to the same pristine PE-MPs used in this study. After ten days of exposure, there was a one- to two-fold increase in mortality after exposure to 80 and 320 mg L^−1^ compared to control. These results, together with those observed in this study, emphasize that particle size, concentration, and exposure act as interacting factors influencing the toxicity of MPs in aquatic organisms, without following a simple or monotonic dose-response relationship.

Exposure to pristine PE-MPs also significantly compromised the reproductive performance of the species (Fig. 3b), with reductions of 7.4 and 5.2 times observed at concentrations of 80 and 140 mg of L^−1^. These reproductive effects are consistent with the results obtained for survival, since the analysis considered the integrated data throughout the entire experimental period, so the observed mortality contributed to the reduction in the total number of neonates produced.

Ogonowski et al. (2016) demonstrated that *D. magna* ingests smaller amounts of food in the presence of MPs when compared to conditions free of these particles. In a complementary way, Giebelhausen and Lampert (2001) showed that, at 20 °C and under similar environmental conditions, the population aptitude of *D. magna* is significantly lower in scenarios of low food availability. In this context, the change in Daphnia’s eating pattern can trigger physiological stress, since the reduction in food intake compromises the body’s energy balance. This energy imbalance is directly reflected in the decrease in the production of neonates observed and, consequently, in the reproductive performance of the species (Martins and Guilhermino 2018).

### Effects of the Mixture of PE-MPs and Imidacloprid

**Fig. 4:**
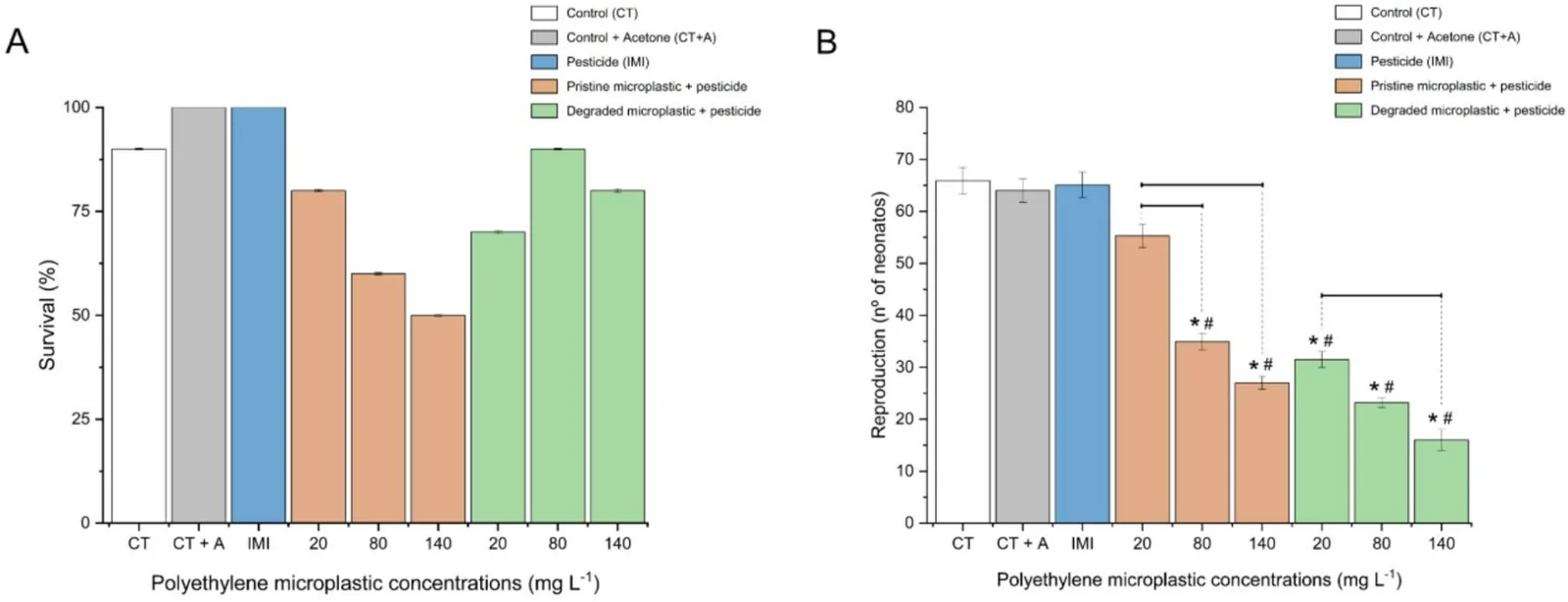
Survival and reproduction of organisms exposed to polyethylene microplastics (PE-MPs) combined with imidacloprid. CT: control; CT + A: control with acetone; IMI: 1.5 mg L^−1^ imidacloprid. (*) Indicates a significant difference compared to the control. # Indicates a significant difference compared to exposure to pesticide alone (*p* < 0.05). Continuous lines indicate significant differences between the concentrations evaluated within the same treatment (*p* < 0.05)

**Fig. 5:**
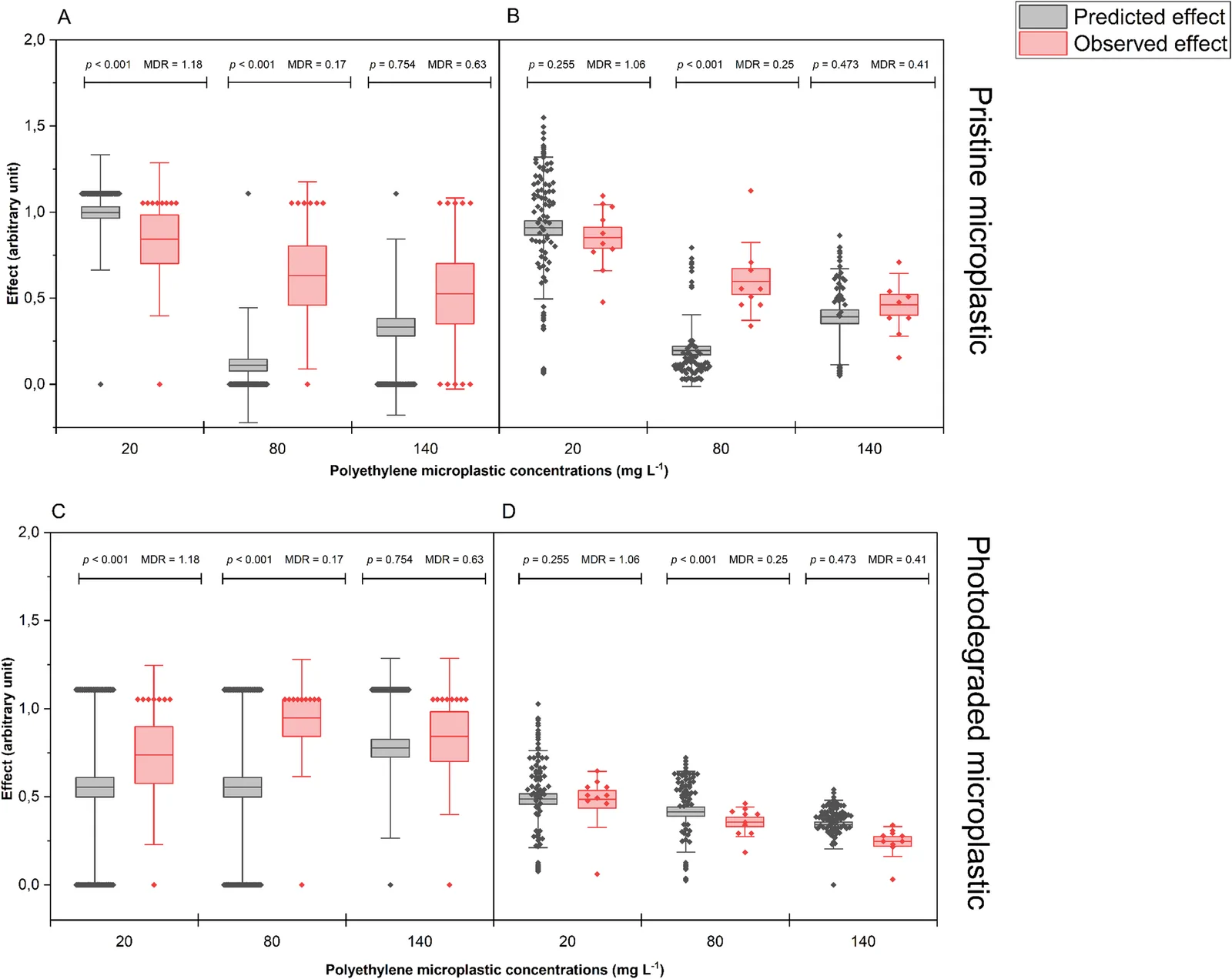
Interaction between pristine and degraded PE-MPs and IMI, evaluated using the MDR method on **a** survival and **b** reproduction of *D. similis* exposed to pristine PE-MPs and on **c** survival and **d** reproduction of *D. similis* exposed to degraded PE-MPs. Upper bars indicate the statistical significance of the deviation between the predicted and observed effects at the evaluated concentrations, with the respective *p* and corresponding MDR values shown above the bars

Figure 4 presents the effects of PE-MPs (pristine and photodegraded) combined with IMI on the survival (Fig. 4a) and reproduction (Fig. 4b) of *D. similis* across the tested concentrations. When considering the combined exposure of pristine PE-MPs and IMI, no lethal effects were observed on survival at any of the tested concentrations (Fig. 4a). To evaluate whether PE-MPs modified IMI toxicity, interactions were assessed using the MDR approach (Fig. 5), which compares observed and expected effects based on single-compound exposures.

At a concentration of 80 mg L^−1^ of pristine MPs in combination with IMI, an antagonistic interaction was evident (Fig. 5a), suggesting an attenuation of PE-MPs toxicity in the presence of IMI. Similar results have been described in the literature, in which organic pesticides, such as glyphosate, chlorpyrifos, and bifenthrin, when combined with polystyrene microplastics, led to a decrease in toxic effects on different aquatic organisms, including the insect *Chironomus tepperi*, the cyanobacterium *Microcystis aeruginosa*, and the alga *Isochrysis galbana* (Garrido et al. 2019; Zhang et al. 2018; Ziajahromi et al. 2019).

At the other concentrations evaluated, a possible additive effect predominated, characterized by the absence of significant interaction between the contaminants. This pattern suggests that, under these conditions, the presence of PE-MPs did not significantly alter the toxicity of the insecticide in the organisms tested. As highlighted by Huang et al. (2021), the toxicity resulting from combined exposure to MPs and organic pollutants in aquatic organisms can vary widely, being strongly conditioned by the physicochemical properties of the particles, the nature of the contaminant, and the sensitivity of the species evaluated. These results reinforce the complexity of the interactions between MPs and IMI and indicate that, in certain exposure scenarios, additive effects may prevail over synergistic or antagonistic responses.

Regarding reproduction (Fig. 4b), significant reductions were observed at all concentrations of pristine PE-MPs except 20 mg L^−1^, with intermediate and high concentrations reducing neonate production by 1.9 and 2.5 times, respectively. The corresponding MDR analysis (Fig. 5b) indicated antagonistic interactions at these concentrations, as the observed effects were lower than those predicted from the individual contaminants.

This pattern may be associated with the neurotoxicity of the insecticide, since studies demonstrate that the feeding of daphnia is mediated by the rhythmic and coordinated movement of filtering appendages, under the control of the nervous system. In this context, exposure to neurotoxic xenobiotics, such as IMI, can interfere with the neuromotor regulation of these appendages, compromising the food filtration process and, consequently, modulating particle ingestion (Villarroel et al. 1999). Such disturbances can result in reduced intake of larger particles, such as PE-MP, which may explain the attenuation of toxicity observed in the present study.

However, prolonged exposures can culminate in starvation mortality, when the energy required to maintain metabolism exceeds the residual feeding rate and energy reserves are depleted. Agatz et al. (2013) demonstrated that the feeding of *D. magna* was significantly inhibited by exposure to IMI (1.83 mg L^−1^) and, by simultaneously varying the food supply and the concentration of the insecticide, as well as modeling the resulting changes in the population growth rate, they showed that the depletion of energy reserves resulting from feeding inhibition was the main cause of the reduction in body size and reproduction. Consistently, Pestana et al. (2010) observed that sublethal exposure to imidacloprid (≤ 8.8 mg L^−1^) reduced feeding rates, growth, and reproduction of *D. magna*. Therefore, although toxicity was attenuated under the combined exposure to PE-MPs and IMI, prolonged exposure to this combination may represent a significant ecological threat.

For photodegraded PE-MPs, combined exposure with IMI also did not induce lethal effects on the survival of *D. similis* (Fig. 4a). The corresponding MDR analysis (Fig. 5c) showed a predominantly additive pattern, characterized by the absence of significant interactions between the contaminants, suggesting that photodegraded PE-MPs did not substantially modify IMI toxicity with respect to survival.

Regarding reproduction (Fig. 4b), photodegraded PE-MPs combined with IMI caused significant reductions, with neonate production being 2.1, 2.8, and 4.1fold lower relative to the control, evidencing a progressive sublethal effect. Despite the expressiveness of these reductions, the MDR analysis (Fig. 5d) indicated a predominantly additive pattern, consistent with the absence of significant interaction between the contaminants. Similar results were reported by Nugnes et al. (2022), who demonstrated that combined exposure to polystyrene microplastics (PS-MPs) and IMI promoted reductions in the offspring of *Ceriodaphnia dubia* that did not differ statistically from those predicted by the isolated effects of each stressor, configuring heteroadditive interactions. Overall, these findings highlight the inherent complexity of interactions between MPs and IMI, since the effects can vary according to the characteristics of the particles, the contaminant, and the exposed organism. In this sense, Mei et al. (2020) highlight that the additive, antagonistic, or synergistic toxic effects between micro or nanoplastic particles and other pollutants can be understood from the role of these particles as carriers of organic compounds, capable of modifying the effective exposure and tissues of the organisms that ingest them.

Furthermore, regarding the effect of degradation on the toxicity of the mixture, it is observed that, at a concentration of 20 mg L^−1^ of PE-MP, photodegradation exerted a significant effect, resulting in a 1.8-fold reduction in the neonate rate. This may be due to the fact that at low concentrations, the greater simultaneous bioavailability of MPs and the contaminants associated with them intensifies direct contact with biological tissues, and may interfere with physiological processes essential to reproduction, such as energy allocation, vitellogenesis, and embryonic development (Chen et al. 2022). Moreover, the degradation process promotes changes on the polymer surface, such as the appearance of cracks and an increase in roughness and surface area, altering toxicity.

In contrast to what was observed for exposure to isolated MPs, the response of *D. similis* to the mixture of MPs with IMI showed a dose-dependent behavior, with a progressive effect on reproduction, especially more intense in the presence of photodegraded MPs. This result may be associated with the fact that the aging of the polymer introduces oxygenated functional groups on its surface, which, as pointed out by Zhang et al. (2024c), can form hydrogen bonds with the amine groups of IMI, intensifying adsorption. In addition, the SEM images showed the presence of cracks, fissures, and irregularities on the surface of the photodegraded MP, expanding the contact area and providing new sorption sites for IMI.

Thus, the results of the present study demonstrate that, although exposure to the mixture with IMI did not cause lethal effects in *D. similis*, the sublethal impacts were significant and ecologically relevant. These effects were reflected in alterations in essential biological functions, such as reproduction, with the potential to compromise the population dynamics of the species in the long term.

## Conclusions

The results of this study demonstrate that the photodegradation of PE-MPs promotes relevant chemical and morphological changes, characterized by the formation of oxygenated groups and increased surface roughness, which intensifies their fragmentation and potential reactivity in the aquatic environment. In ecotoxicological terms, it was found that pristine PE-MPs affect the survival of *D. similis* more immediately, while photodegraded ones exert sublethal impacts with a toxicity profile dependent on the state of degradation.

Treatments involving pristine PE-MPs showed the most pronounced effects, possibly due to the induction, by the MPs, of a false sense of satiety and favors the occurrence of mechanical lesions in the intestinal walls, triggering physiological stress. In turn, the interaction between IMI and PE-MPs revealed both additive effects and significant antagonistic interactions, in which IMI attenuated the toxicity of the mixture by potentially reducing the ingestion of PE-MPs by *D. similis*. Taken together, these results reinforce that the simultaneous presence of PE-MPs and pesticides can compromise essential biological functions, even in the absence of immediate lethal effects, indicating a potential risk to the maintenance of populations in freshwater ecosystems in the long term.

Furthermore, the results of the present study reinforce that the assessment of ecotoxicological effects associated with MPs cannot be dissociated from either the state of degradation of the material or its interactions with other chemical contaminants. Although the most pronounced effects were observed for pristine MPs, the data indicate that, in combined exposures, the state of degradation can act as a modulating factor of the effects, especially on the reproduction of *D. similis*. These findings highlight the potential of PE-MPs to compromise the population dynamics of aquatic organisms, with relevant implications for the stability and functioning of aquatic ecosystems.

Thus, this study reinforces the need for integrated ecotoxicological approaches that consider chronic exposures, contaminant mixtures, and environmentally realistic scenarios for a more robust and accurate assessment of the impacts of MPs on aquatic ecosystems.

## Supplementary Information

Below is the link to the electronic supplementary material.

Supplementary Material 1

## Data Availability

The data supporting the findings of this study are presented in this article and its supplementary material.
