# Supplementary material for: Integrated Study of Polyethylene Microplastic Degradation and Its Interaction with the Insecticide Imidacloprid: Chronic Toxicity to Daphnia similis
Source: Arch Environ Contam Toxicol. 2026 Sep 2;91(2):16. doi: 10.1007/s00244-026-01213-5 (PMC13538188; doi:10.1007/s00244-026-01213-5)
Supplement: Supplementary file 1 [file 244_2026_1213_MOESM1_ESM.docx]

**Integrated Study of Polyethylene Microplastic Degradation and Its Interaction with the Insecticide Imidacloprid: Chronic Toxicity to *Daphnia similis***

Maria Eduarda Kiihl ^a*^, Thandy Junio da Silva Pinto ^b^, Mariana Amaral Dias ^b^, Cassiana Carolina Montagner ^b^, Evaldo Luiz Gaeta Espíndola ^a^, David Silva Alexandre ^a^

^a^ Laboratory of Ecotoxicology and Applied Ecology, Department of Hydraulic and Sanitation, São Carlos School of Engineering, University of São Paulo, São Carlos, SP, Brazil

^b^ University of Campinas (UNICAMP), Institute of Chemistry, Campinas, São Paulo, Brazil

**Orcid:** Maria Eduarda Kiihl: 0009-0000-4564-4561 ([mariakiihl@usp.br](mailto:mariakiihl@usp.br)); Thandy Junio da Silva Pinto: 0000-0003-1640-8329 (thandyjuniosilva@gmail.com); Mariana Amaral Dias: 0000-0003-0639-5288 (marimari.dias@gmail.com); Cassiana Carolina Montagner: 0000-0002-6475-5969 (ccmonta@unicamp.br); Evaldo Luiz Gaeta Espíndola: 0000-0003-1700-9661 (elgaeta@sc.usp.br); David Silva Alexandre: 0000-0002-9305-7895 (davidalexandre@usp.br)

*** Corresponding author:**

Maria Eduarda Kiihl

Laboratory of Ecotoxicology and Applied Ecology, São Carlos Engineering School, University of São Paulo, Av. Trabalhador São Carlense, 400, São Carlos 13560-970, Brazil

e-mail: mariakiihl@usp.br

**Figure S1** Microscopic images of pristine PE-MPs

**Figure S2** Histogram of the particle size distribution of pristine e degraded microplastics

**Figure S3** Temperature variation in the accelerated weathering chamber throughout the degradation period

**Figure S4** Presence of PE-MP particles in the digestive tract of *D. similis after* 21 days of exposure to the control and concentrations of 20 and 80 mg L^-1^, respectively

**Table 1** Estimation of the particle concentration range (particles m^-3^) in the selected treatments based on (Leusch and Ziajahromi, 2021)

| mg L ^-1^ | part m ^-3^ | | | | | | |
| --- | --- | --- | --- | --- | --- | --- | --- |
|  | 5 to 10 µm | 10 to 15 µm | 15 to 20 µm | 20 to 25 µm | 25 to 30 µm | 30 to 35 µm | 35 to 40 µm |
| 20 | 9.63 x 10^10^ | 2.08 x 10^10^ | 7.58 x 10^9^ | 3.57 x 10^9^ | 1.95 x 10^9^ | 1.18 x 10^9^ | 7.71 x 10^8^ |
| 80 | 3.85 x 10^11^ | 8.32 x 10^10^ | 3.03 x 10^10^ | 1.43 x 10^10^ | 7.82 x 10^9^ | 4.73 x 10^9^ | 3.08 x 10^9^ |
| 140 | 6.74 x 10^11^ | 1.46 x 10^11^ | 5.31 x 10^10^ | 2.50 x 10^10^ | 1.37 x 10^10^ | 8.29 x 10^9^ | 5.39 x 10^9^ |
| mg L ^-1^ | **part L ^-1^** | | | | | | |
|  | 5 to 10 µm | 10 to 15 µm | 15 to 20 µm | 20 to 25 µm | 25 to 30 µm | 30 to 35 µm | 35 to 40 µm |
| 20 | 9.63 x 10^7^ | 2.08 x 10^7^ | 7.58 x 10^6^ | 3.57 x 10^6^ | 1.95 x 10^6^ | 1.18 x 10^6^ | 7.71 x 10^5^ |
| 80 | 3.85 x 10^8^ | 8.32 x 10^7^ | 3.03 x 10^7^ | 1.43 x 10^7^ | 7.82 x 10^6^ | 4.73 x 10^6^ | 3.08 x 10^6^ |
| 140 | 6.74 x 10^8^ | 1.46 x 10^8^ | 5.31 x 10^7^ | 2.50 x 10^7^ | 1.37 x 10^7^ | 8.29 x 10^6^ | 5.39 x 10^6^ |

| mg L ^-1^ | part m ^-3^ | | | | | | |
| --- | --- | --- | --- | --- | --- | --- | --- |
|  | 40 to 45 µm | 45 to 50 µm | 50 to 55 µm | 55 to 60 µm | 60 to 65 µm | 65 to 70 µm | 70 to 75 µm |
| 20 | 5.29 x 10^8^ | 3.79 x 10^8^ | 2.81 x 10^8^ | 2.14 x 10^8^ | 1.66 x 10^8^ | 1.32 x 10^8^ | 1.07 x 10^8^ |
| 80 | 2.11 x 10^9^ | 1.51 x 10^9^ | 1.12 x 10^9^ | 8.55 x 10^8^ | 6.66 x 10^8^ | 5.29 x 10^8^ | 4.27 x 10^8^ |
| 140 | 3.70 x 10^9^ | 2.65 x 10^9^ | 1.96 x 10^9^ | 1.49 x 10^9^ | 1.16 x 10^9^ | 9.25 x 10^8^ | 7.46 x 10^8^ |
| mg L ^-1^ | **part L ^-1^** | | | | | | |
|  | 40 to 45 µm | 45 to 50 µm | 50 to 55 µm | 55 to 60 µm | 60 to 65 µm | 65 to 70 µm | 70 to 75 µm |
| 20 | 5.29 x 10^5^ | 3.79 x 10^5^ | 2.81 x 10^5^ | 2.14 x 10^5^ | 1.66 x 10^5^ | 1.32 x 10^5^ | 1.07 x 10^5^ |
| 80 | 2.11 x 10^6^ | 1.51 x 10^6^ | 1.12 x 10^6^ | 8.55 x 10^8^ | 6.66 x 10^5^ | 5.29 x 10^5^ | 4.27 x 10^5^ |
| 140 | 3.70 x 10^6^ | 2.65 x 10^6^ | 1.96 x 10^6^ | 1.49 x 10^6^ | 1.16 x 10^6^ | 9.25 x 10^5^ | 7.46 x 10^5^ |
